# Adverse Benzo[*a*]pyrene Effects on Neurodifferentiation Are Altered by Other Neurotoxicant Coexposures: Interactions with Dexamethasone, Chlorpyrifos, or Nicotine in PC12 Cells

**DOI:** 10.1289/ehp.1306528

**Published:** 2013-04-19

**Authors:** Theodore A. Slotkin, Jennifer Card, Frederic J. Seidler

**Affiliations:** Department of Pharmacology and Cancer Biology, Duke University Medical Center, Durham, North Carolina, USA

**Keywords:** benzo[*a*]pyrene, chlorpyrifos, dexamethasone, neurodifferentiation, nicotine, organophosphate pesticides, PAHs, PC12 cells, polycyclic aromatic hydrocarbons

## Abstract

Background: Polycyclic aromatic hydrocarbons are suspected developmental neurotoxicants, but human exposures typically occur in combination with other neurotoxic contaminants.

Objective and Methods: We explored the effects of benzo[*a*]pyrene (BaP) on neurodifferentiation in PC12 cells, in combination with a glucocorticoid (dexamethasone, used in preterm labor), an organophosphate pesticide (chlorpyrifos), or nicotine.

Results: In cells treated with BaP alone, the transition from cell division to neurodifferentiation was suppressed, resulting in increased cell numbers at the expense of cell growth, neurite formation, and development of dopaminergic and cholinergic phenotypes. Dexamethasone enhanced the effect of BaP on cell numbers and altered the impact on neurotransmitter phenotypes. Whereas BaP alone shifted differentiation away from the cholinergic phenotype and toward the dopaminergic phenotype, the addition of dexamethasone along with BaP did the opposite. Chlorpyrifos coexposure augmented BaP inhibition of cell growth and enhanced the BaP-induced shift in phenotype toward a higher proportion of dopaminergic cells. Nicotine had no effect on BaP-induced changes in cell number or growth, but it synergistically enhanced the BaP suppression of differentiation into both dopaminergic and cholinergic phenotypes equally.

Conclusion: Our results indicate that, although BaP can act directly as a developmental neurotoxicant, its impact is greatly modified by coexposure to other commonly encountered neurotoxicants from prenatal drug therapy, pesticides, or tobacco. Accordingly, neurodevelopmental effects attributable to polycyclic aromatic hydrocarbons may be quite different depending on which other agents are present and on their concentrations relative to each other.

Exposures to polycyclic aromatic hydrocarbons (PAHs) are ubiquitous, given their presence in combustion products of all kinds, including diesel exhaust, broiled foods, and smoke arising from wood, coal, or tobacco. Although most studies of the adverse effects of PAHs center around their properties as carcinogens, recent reports have indicated that these agents are also developmental neurotoxicants, potentially contributing to the substantial increase in the incidence of neurobehavioral disorders (Grandjean and Landrigan 2006). Epidemiological studies show a relationship between fetal PAH exposure, head circumference, and cognitive performance (Perera et al. 2003, 2006), but few animal studies have explored whether these agents directly affect brain development. Benzo[*a*]pyrene (BaP), a PAH prototype, produces behavioral deficits in adults (Saunders et al. 2002) and, after fetal or neonatal exposure, leads to persistent anomalies in cognitive performance, anxiety-related behaviors, hippocampal function, and neurochemical indices of cerebral activity (Chen et al. 2012; Hood et al. 2000; Wormley et al. 2004). BaP exposure of neuronal cells in culture interferes with neurodifferentiation (Brown et al. 2007; Slotkin and Seidler 2009), indicating that PAHs act directly as developmental neurotoxicants, exclusive of endocrine disruption or other systemic effects in the maternal–fetal unit or the newborn. We recently showed that BaP slows the ability of differentiating neuronotypic cells to exit the mitotic cycle and to initiate neurodifferentiation, resulting in increases in cell number at the expense of cell growth, neurite formation, and development of neurotransmitter phenotypes (Slotkin and Seidler 2009). Such direct interference with neural cell differentiation could readily explain the observed correlation of prenatal PAH exposures in humans to behavioral dysfunction (Perera et al. 2005, 2006).

One major difference between laboratory and human PAH studies is that although basic research tends to focus on exposures to single agents, humans are simultaneously exposed to other developmental neurotoxicants along with the PAHs, such as tobacco smoke and pesticides (Perera et al. 2005). In the present study, we examined whether the direct effects of PAHs on neuronal development are modified by simultaneous exposure to other commonly encountered neurotoxicants. Specifically, we looked at the combination of BaP with a glucocorticoid (dexamethasone), an organophosphate pesticide (chlorpyrifos), and nicotine. Each of these secondary agents has been well-studied for developmental neurotoxicity *in vivo* and *in vitro*, and they all represent major human exposure hazards. Glucocorticoids are the consensus treatment for preterm labor occurring between 24 and 34 weeks of gestation in order to prevent respiratory distress syndrome (Gilstrap et al. 1995); currently, 1 of every 10 newborns in the United States has undergone this treatment (Matthews et al. 2002). Organophosphates represent nearly 50% of worldwide insecticide use, and exposure of the human population is virtually ubiquitous (Casida and Quistad 2004). Nicotine coexposure with PAHs is common because of the presence of both in cigarette smoke, whether from active maternal smoking or from second- and third-hand exposure (Hoh et al. 2012; Perera et al. 2005).

For our study, we used PC12 cells, a well-characterized model for neurodifferentiation (Teng and Greene 1994), with protocols established previously for characterizing developmental neurotoxicity (Qiao et al. 2001, 2003, 2005; Slotkin et al. 2007a, 2007b, 2008; Song et al. 1998). Effects on cell number were determined by measuring DNA content, because each neuronotypic cell contains only a single nucleus (Winick and Noble 1965). Cell size and membrane outgrowth associated with the formation of neurites were assessed by measurements of cell proteins (total protein/DNA ratio, membrane protein/DNA ratio, and membrane protein/total protein ratio). Finally, we assayed tyrosine hydroxylase (TH) and choline acetyltransferase (ChAT), the two enzymes that delineate differentiation into the dopaminergic (TH) and cholinergic (ChAT) phenotypes that are the distinctive fate of PC12 cells (Teng and Greene 1994).

## Materials and Methods

*Cell cultures*. Because of the clonal instability of the PC12 cell line (Fujita et al. 1989), we performed experiments on cells that had undergone fewer than five passages. As described previously (Qiao et al. 2003; Song et al. 1998), PC12 cells (American Type Culture Collection CRL-1721, obtained from the Duke Comprehensive Cancer Center, Durham, NC) were seeded onto poly-d-lysine–coated plates in RPMI-1640 medium (Sigma Chemical Co., St. Louis, MO) supplemented with 10% horse serum (Sigma), 5% fetal bovine serum (Sigma), and 50 µg/mL penicillin streptomycin (Invitrogen, Carlsbad, CA). Incubations were carried out with 5% CO_2_ at 37°C, standard conditions for PC12 cells. To initiate neurodifferentiation (Jameson et al. 2006b; Slotkin et al. 2007b; Teng and Greene 1994), the medium was changed to include 50 ng/mL of 2.5 S murine nerve growth factor (Promega Corporation, Madison, WI); each culture was examined under a microscope to verify the outgrowth of neurites.

Toxicant exposures were all started simultaneously with the addition of nerve growth factor, so that the toxicants were present throughout neurodifferentiation. For BaP (Sigma) exposure, we chose two concentrations based on earlier studies with this model (Slotkin and Seidler 2009), one just at the threshold for effects (1 µM) and the second one a higher concentration showing robust effects (10 µM); these concentrations are similar to those shown *in vivo* to produce lasting neurochemical and neurobehavioral effects after early-life exposures (Brown et al. 2007; Chen et al. 2012). Concentrations for the other agents [dexamethasone (0.1 µM; Sigma), chlorpyrifos (30 µM; Chem Service, West Chester, PA), and nicotine bitartrate (10 µM; Sigma)] were also based on earlier work and were chosen to be just at the threshold for effects on cell number and growth in order to allow the detection of interactions with BaP (Abreu-Villaça et al. 2005; Jameson et al. 2006a; Qiao et al. 2003; Slotkin et al. 2012; Song et al. 1998). For dexamethasone, we conducted additional studies using a higher concentration (1 µM). Because of the limited water solubility of BaP and chlorpyrifos, these agents were dissolved in dimethylsulfoxide (DMSO, Sigma; final concentration 0.1%), which was also added to all the samples regardless of treatment; this concentration of DMSO has no effect on PC12 cell growth or differentiation (Qiao et al. 2001; Song et al. 1998). The medium containing nerve growth factor and test substances was changed every 48 hr; assays were carried out after 6 days of exposure.

*Assays.* Cells were harvested and washed, and the DNA and protein fractions were isolated and analyzed as described previously (Slotkin et al. 2007b). Measurements of DNA, total protein, and membrane protein were used as biomarkers for cell number, cell growth, and neurite growth (Qiao et al. 2003; Song et al. 1998). Because the DNA per cell is constant, cell growth entails an obligatory increase in the total protein per cell (protein/DNA ratio) as well as membrane protein per cell (membrane protein/DNA ratio). If cell growth represents simply an increase in the perikaryal area, the ratio of membrane protein to total protein would fall in parallel with the decline in the surface/volume ratio (volume increases with the cube of the perikaryal radius, whereas surface area increases with the square of the radius). However, when neurites are formed as a consequence of neurodifferentiation, this produces a specific increase in the ratio. Each of these biomarkers has been validated in prior studies by direct measurement of cell number (Powers et al. 2010; Roy et al. 2005), perikaryal area (Roy et al. 2005), and neurite formation (Das and Barone 1999; Howard et al. 2005; Song et al. 1998). To assess neurodifferentiation into dopamine and acetylcholine phenotypes, we assayed the activities of TH and ChAT, respectively, using established techniques (Jameson et al. 2006a, 2006b).

*Data analysis.* Each study was performed using two to five separate batches of cells, with three or four independent cultures for each treatment in each batch; each batch of cells comprised a separately prepared, frozen and thawed passage. Results are presented as mean ± SE, with treatment comparisons carried out by analysis of variance (ANOVA; with data log-transformed when variance was heterogeneous or if comparisons were based on proportional changes) followed by Fisher’s protected least significant difference test for post hoc comparisons of individual treatments. Each treatment paradigm involved an initial three-factor ANOVA, with factor 1 the BaP concentration, factor 2 the concentration of second agent (dexamethasone, chlorpyrifos, or nicotine), and factor 3 the cell batch. In each case, we found that the treatment effects were the same across the different batches of cells, although the absolute values differed from batch to batch. Accordingly, we normalized the results across batches prior to combining them for presentation. Significance was assumed at *p* < 0.05.

The experimental design required two different ways of considering the treatment variables. To characterize the effects of BaP alone, the second agent alone, or the combined treatment versus controls or versus each other, all of the treatment groups were first considered as a one-dimensional factor in the statistical design. In this formulation, each treatment can be compared to the control group or to any of the other treatments. Then, to determine whether the effects of BaP and the second agent were interactive, the treatment factors were changed to a two-dimensional design (with factor 1 being BaP and factor 2 the second agent). In this formulation, synergistic, less-than-additive, or antagonistic effects would appear as significant interactions between the two treatment dimensions, whereas simple, additive effects would not show significant interactions. For example, although the one-factor arrangement of the data can show that a combined exposure might be worse than either exposure alone, the two-factor arrangement enables us to determine whether the worsened effect represents the additive effects of the two agents or whether the combination gives a response that is greater or less than the predicted additive value.

## Results

**Figure 1:**
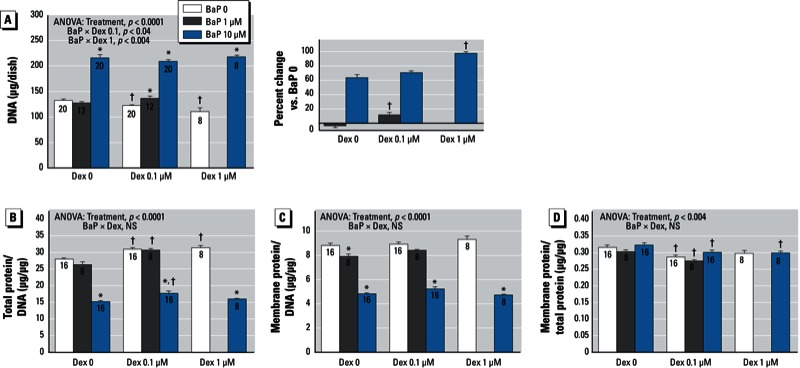
Effects of BaP in combination with dexamethasone (Dex) on indices of cell number and cell growth. (*A*) DNA. (*B*) Total protein/DNA ratio. (*C*) Membrane protein/DNA ratio. (*D*) Membrane protein/total protein. Where there was a significant interaction, the percent change caused by BaP relative to the corresponding BaP 0 group for each dexamethasone concentration is shown on the right (*A*). Data represent means ± SEs of the number of determinations shown within bars. ANOVAs for the main effects of treatment and interactions of BaP with Dex are shown at the top of each panel. We did not evaluate the combination of 1 µM BaP and 1 µM Dex. NS, not significant. *p < 0.05 compared with the corresponding BaP 0 group. ^†^p < 0.05 compared with the corresponding Dex 0 group.

*BaP and dexamethasone*. In agreement with earlier results (Slotkin and Seidler 2009), exposure to BaP alone during neurodifferentiation produced an elevation in the total of number of cells, as monitored by DNA content (Figure 1A), at the expense of cell enlargement, as evidenced by decrements in the total protein/DNA ratio (Figure 1B) and membrane protein/DNA ratio (Figure 1C). BaP alone did not produce a significant change in the membrane protein/total protein ratio (Figure 1D); because smaller cells have an elevated ratio, the lack of change in this parameter—combined with the drop in total protein/DNA (smaller cells)—connotes interference with the neurite formation that accompanies neurodifferentiation (Slotkin and Seidler 2009). In the absence of BaP, dexamethasone produced a significant decrease in cell numbers (Figure 1A). Accordingly, if the two treatments were simply additive, dexamethasone would be expected to reduce the effect of BaP on DNA content. Instead, it augmented it. At the low concentration of dexamethasone (0.1 µM), 1 µM BaP produced a significant increase in cell numbers, whereas the same concentration was ineffective in the absence of dexamethasone. The increase evoked by 10 µM BaP in the presence of dexamethasone remained just as high as before; superimposed on the reduced baseline values caused by dexamethasone alone, the effects of BaP were thus synergistically enhanced (Figure 1A, right).

Dexamethasone treatment alone had the opposite effect on cell growth from that of BaP, evidenced by an increase in total protein/DNA (Figure 1B). When the two treatments were combined, the net outcome reflected these opposing actions, representing simple additive effects (no interaction of BaP × dexamethasone). Dexamethasone had little or no effect on the membrane protein/DNA ratio, and again the combination showed simple additive actions of the two agents (Figure 1C). Because dexamethasone increased cell size without changing the membrane protein concentration, the membrane protein/total protein ratio decreased, reflecting impaired neurite formation (Figure 1D), which is in agreement with earlier findings (Jameson et al. 2006a); again, this effect showed simple additivity with BaP.

**Figure 2:**
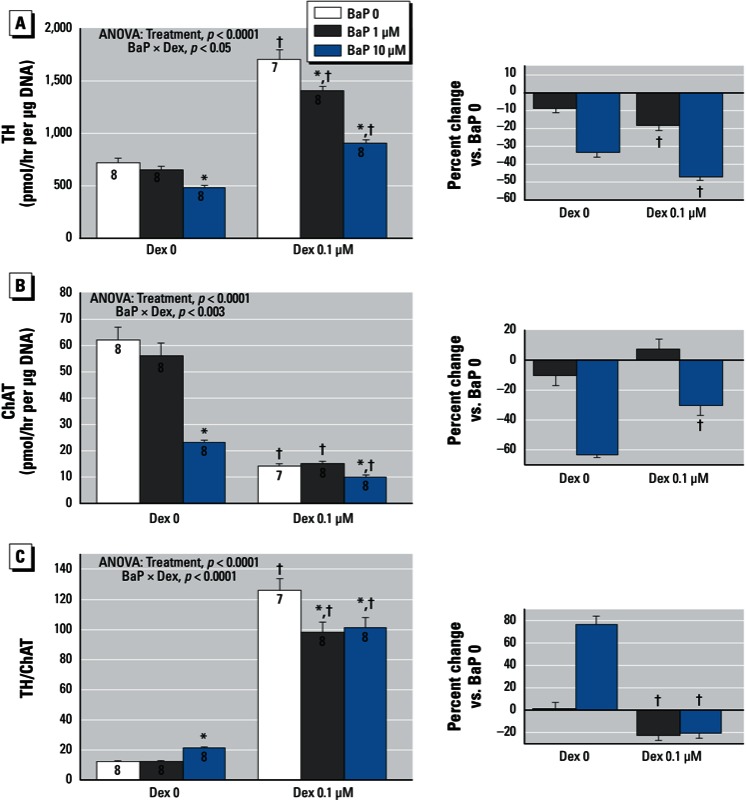
Effects of BaP in combination with dexamethasone (Dex) on neurodifferentiation into dopaminergic and cholinergic phenotypes. (*A*) TH (tyrosine hydroxylase). (*B*) ChAT (choline acetyltransferase). (*C*) TH/ChAT ratio. The right hand panels show the percent change caused by BaP relative to the corresponding BaP 0 group for each Dex concentration. Data represent means ± SEs of the number of determinations shown within bars. ANOVAs for the main effects of treatment and interactions of BaP with Dex are shown at the top of each panel. NS, not significant. *p < 0.05 compared with the corresponding BaP 0 group. ^†^p < 0.05 compared with the corresponding Dex 0 group.

Individually, BaP and dexamethasone had opposite effects on TH activity (Figure 2A). By itself, BaP evoked a reduction in TH, whereas dexamethasone alone produced a substantial increase. In the presence of dexamethasone, BaP showed an enhanced ability to reduce TH, reflecting a synergistic interaction of the two treatments (Figure 2A, right). We observed a different pattern for effects on ChAT activity (Figure 2B). BaP alone produced a large decrease, as did dexamethasone; the combined treatment also showed a decrement, but it was distinctly less than would be expected from simple additive effects of the two treatments (Figure 2B, right). Accordingly, the phenotypic outcome, assessed by the TH/ChAT ratio, was completely reversed by the double treatment (Figure 2C). Either BaP or dexamethasone alone elevated the ratio, reflecting a shift from the cholinergic to the dopaminergic phenotype. However, in the presence of dexamethasone, BaP reduced the ratio, an effect opposite to that seen with BaP alone.

**Figure 3:**
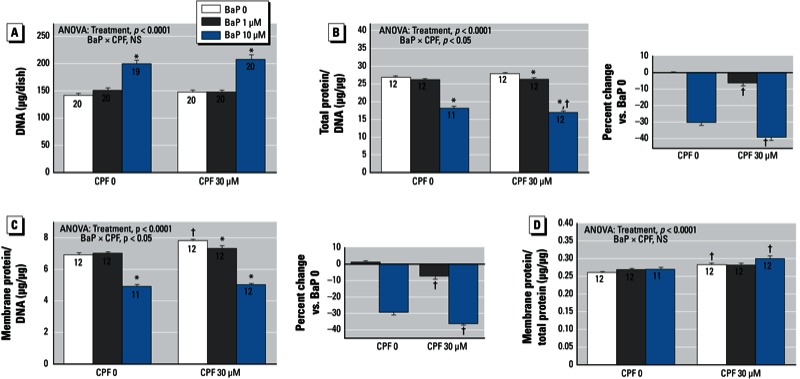
Effects of BaP in combination with chlorpyrifos (CPF) on indices of cell number and cell growth. (*A*) DNA. (*B*) Total protein/DNA ratio. (*C*) Membrane protein/DNA ratio. (*D*) Membrane protein/total protein. Where there was a significant interaction, the percent change caused by BaP relative to the corresponding BaP 0 group for each CPF concentration is shown on the right. Data represent means ± SEs of the number of determinations shown within bars. ANOVAs for the main effects of treatment and interactions of BaP with CPF are shown at the top of each panel; NS, not significant. *p < 0.05 compared with the corresponding BaP 0 group. ^†^p < 0.05 compared with the corresponding CPF 0 group.

*BaP and chlorpyrifos*. In contrast to the combination of BaP plus dexamethasone, cotreatment of BaP plus chlorpyrifos produced interactions primarily involving cell growth parameters rather than cell numbers. Chlorpyrifos did not alter the effect of BaP on DNA content (Figure 3A), but it did shift the response of both the total protein/DNA ratio (Figure 3B) and the membrane protein/DNA ratio (Figure 3C), enhancing the reduction caused by BaP (significant BaP × chlorpyrifos interaction for both ratios). Significant enhancement of the BaP effect was obtained at either the low or high concentration of BaP (Figure 3B, right; Figure 3C, right). We observed a small but significant increase in the membrane protein/total protein ratio in the chlorpyrifos groups, regardless of whether BaP was included, with no interaction between the two treatments (Figure 3D).

**Figure 4:**
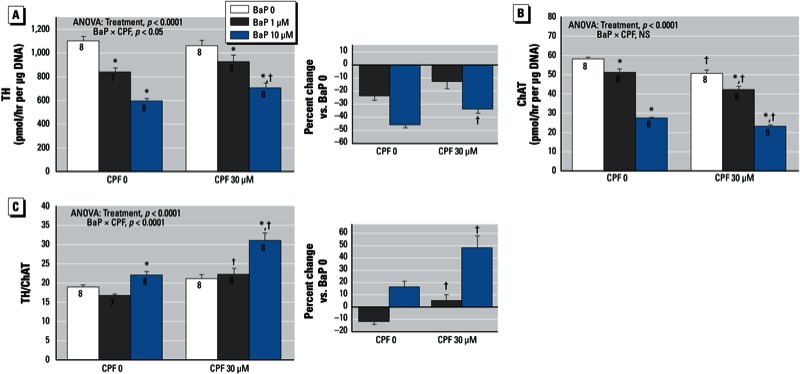
Effects of BaP in combination with chlorpyrifos (CPF) on neurodifferentiation into dopaminergic and cholinergic phenotypes. (*A*) TH (tyrosine hydroxylase). (*B*) ChAT (choline acetyltransferase). (*C*) TH/ChAT ratio. Where there was a significant interaction, the percent change caused by BaP relative to the corresponding BaP 0 group for each CPF concentration is shown on the right. Data represent means ± SEs of the number of determinations shown within bars. ANOVAs for the main effects of treatment and interactions of BaP with CPF are shown at the top of each panel. NS, not significant. *p < 0.05 compared with the corresponding BaP 0 group. ^†^p < 0.05 compared with corresponding CPF 0 group.

Chlorpyrifos altered the ability of BaP to suppress neurodifferentiation. For TH activity, the combined treatment (BaP plus chlorpyrifos) showed a smaller BaP-induced decrement than that obtained with BaP alone, which was reflected in a significant BaP × chlorpyrifos interaction (Figure 4A). By itself, chlorpyrifos reduced ChAT, but the interaction with BaP was purely additive (no interaction); thus, the combined treatment produced an even greater reduction in ChAT than did treatment with BaP or chlorpyrifos alone (Figure 4B). With the combined treatment, the smaller decrease in TH and the greater deficit in ChAT produced a larger elevation of the TH/ChAT ratio than that obtained with either treatment alone (Figure 4C), a synergistic enhancement that reflected greater-than-additive effects (Figure 4C, right).

**Figure 5:**
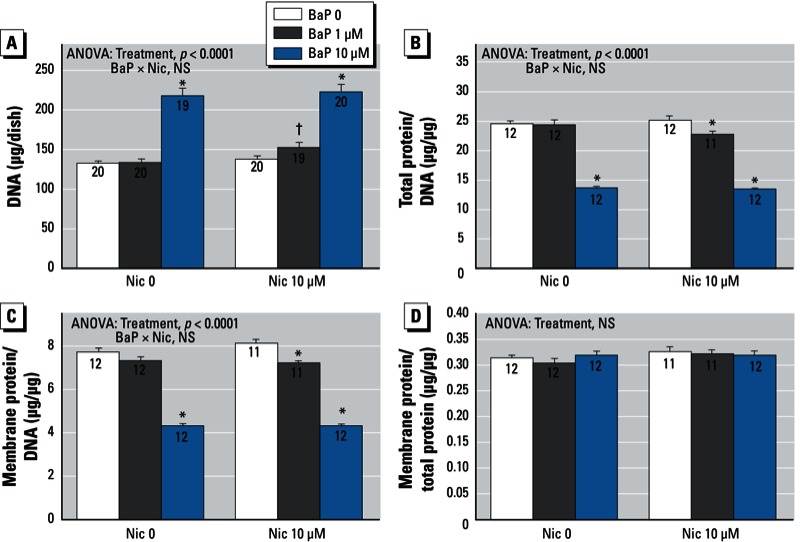
Effects of BaP in combination with nicotine (Nic) on indices of cell number and cell growth. (*A*) DNA. (*B*) Total protein/DNA ratio. (*C*) Membrane protein/DNA ratio. (*D*) Membrane protein/total protein. Data represent means ± SEs of the number of determinations shown within bars. ANOVAs for the main effects of treatment and interactions of BaP with nicotine are shown at the top of each panel. NS, not significant. *p < 0.05 compared with the corresponding BaP 0 group. ^†^p < 0.05 compared with the corresponding Nic 0 group.

**Figure 6:**
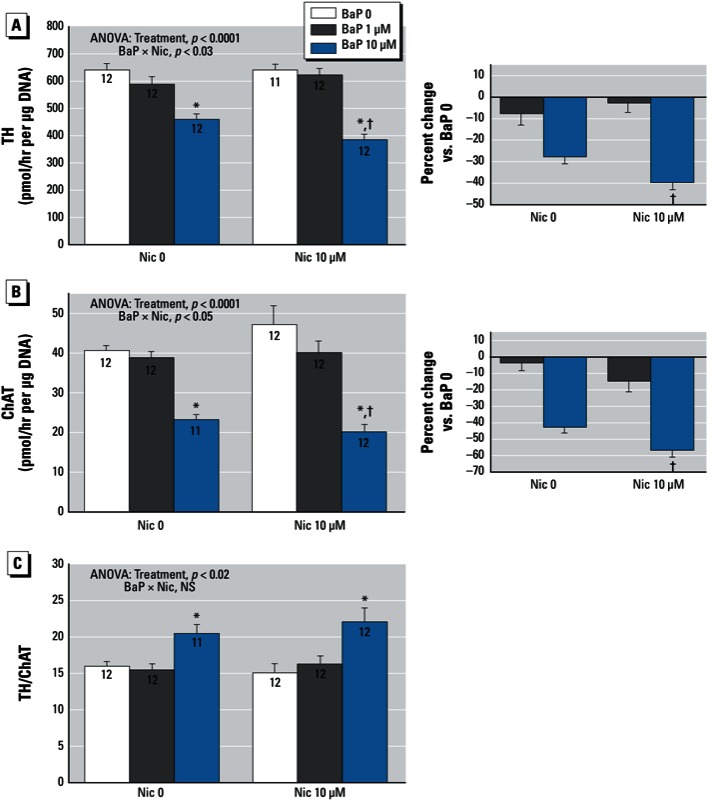
Effects of BaP in combination with nicotine (Nic) on neurodifferentiation into dopaminergic and cholinergic phenotypes. (*A*) TH (tyrosine hydroxylase). (*B*) ChAT (choline acetyltransferase). (*C*) TH/ChAT ratio. Where there was a significant interaction, the percent change caused by BaP relative to the corresponding BaP 0 group for each Nic concentration is shown on the right. Data represent means ± SEs of the number of determinations shown within bars. ANOVAs for the main effects of treatment and inter­actions of BaP with nicotine are shown at the top of each panel. NS, not significant. *p < 0.05 compared with the corresponding BaP 0 group. ^†^p < 0.05 compared with the corresponding Nic 0 group.

*BaP and nicotine*. In contrast to the other agents, 10 µM nicotine did not alter the effect of BaP on any of the parameters of cell number or growth (Figure 5). Nevertheless, it did influence the phenotypic outcome. For TH activity, nicotine enhanced the BaP-induced suppression, resulting in a larger deficit (Figure 6A); the effect showed a significant BaP × nicotine interaction, indicating synergistic effects (Figure 6A, right). The same pattern was observed for ChAT, namely an enhanced effect of BaP in the presence of nicotine, resulting in greater-than-additive reductions in activity (Figure 6B). Because the synergistic effect of the combined treatment was equivalent for both TH and ChAT, the shift toward the dopaminergic phenotype and away from the cholinergic phenotype (increased TH/ChAT ratio) was equivalent for BaP alone and for BaP plus nicotine (Figure 6C).

## Discussion

The major finding of this study is that the ability of BaP to interfere with neurodifferentiation is greatly modified by coexposure to other common toxicants. For some end points, the effects of the secondary agent plus BaP are opposite to those of BaP alone. This implies that, for environmental exposures, the observed outcome for BaP is likely to depend on the nature and concentration of other neurotoxicants to which the fetus or neonate has been exposed.

By itself, BaP produced effects entirely consistent with impaired neurodifferentiation (Slotkin and Seidler 2009). Upon addition of nerve growth factor, PC12 cells begin to exit the mitotic cycle and differentiate into dopaminergic and cholinergic neuronal phenotypes (Teng and Greene 1994). BaP prolongs the period of mitotic activity, resulting in elevated cell numbers, at the expense of cell growth and differentiation (Slotkin and Seidler 2009). Here, this was shown by an elevation in DNA content (more cells), along with reductions in indices of cell enlargement, neurite formation, and emergence of neurotransmitter phenotypes, with the latter showing a greater impairment for acetylcholine (ChAT) than for dopamine (TH). Coexposure with dexamethasone enhanced the effects of BaP. Although treatment with dexamethasone alone reduced DNA content, the addition of BaP produced a synergistic increase above the baseline effect of dexamethasone, which was proportionally larger than that seen with BaP alone. Even more striking, in the presence of dexamethasone, BaP evoked greater suppression of TH activity, whereas it had a lesser effect on ChAT. Thus, the combined exposure to BaP and dexamethasone reversed the impact of BaP on phenotype: The TH/ChAT ratio was increased by BaP alone but was decreased by BaP plus dexamethasone. Accordingly, dexamethasone completely shifted the impact of BaP on neurodifferentiation.

Although chlorpyrifos also altered the response to BaP, the outcomes were entirely different from those seen with dexamethasone. Rather than augmenting the effects on cell number, chlorpyrifos enhanced BaP inhibition of cell growth. At the same time, chlorpyrifos reduced the ability of BaP to impair TH emergence; consequently, the impact on neuronal differentiation was to promote the dopaminergic phenotype at the expense of the cholinergic phenotype, to an even greater extent than was seen with either agent alone. This produced an even greater increase in the TH/ChAT ratio, exactly the opposite outcome from that observed when BaP was combined with dexamethasone. It is notable that these combined effects of chlorpyrifos and BaP reflect direct targeting of neurodifferentiation, rather than effects secondary to cholinesterase inhibition; PAHs and chlorpyrifos show additive inhibitory effects on cholinesterase (Jett et al. 1999), whereas we found that chlorpyrifos interfered with the effect of BaP on TH.

Nicotine coexposure produced yet a third set of outcomes for the effects of BaP on neurodifferentiation. Nicotine did not affect the ability of BaP to increase cell numbers or impair growth parameters, but it produced synergistic effects on suppression of both the dopaminergic and cholinergic phenotypes. In this case, the interaction was equally targeted toward TH and ChAT: Although both neurotransmitter subtypes showed deficits, the combined exposure produced no further shift in phenotypic preference compared with BaP alone.

There are obvious limitations inherent in any *in vitro* model of developmental neurotoxicity, as described previously (Coecke et al. 2007; Qiao et al. 2001; Song et al. 1998), but it is worth repeating the major points here. The main purpose of *in vitro* models is to assess direct effects of toxicants, allowing for dissection of cause-and-effect relationships that cannot readily be studied *in vivo*. Thus, the first limitation is that cell culture models lack the ability to detect more complex neurodevelopmental events involved in brain assembly, including cell-to-cell interactions and architectural modeling of brain regions. Second, *in vitro* exposures typically involve treatments over a period of hours, whereas *in vivo* exposures encompass much more extended exposure periods. Third, transformed cell lines, such as PC12 cells, usually are less sensitive to toxicants than are primary neurons. All these factors mean that it is difficult to extrapolate relevant *in vivo* concentrations of toxicants from *in vitro* results alone, and typically, the concentrations required for a given effect *in vitro* will be substantially higher than those required for parallel effects *in vivo* (Coecke et al. 2007). Nevertheless, the BaP concentrations used here do correspond to doses required for adverse effects in developing rats (Brown et al. 2007; Chen et al. 2012).

In the present study, we chose the PC12 line for specific reasons. The primary effect of BaP is to delay the transition from cell replication to neurodifferentiation (Slotkin and Seidler 2009). Primary neurons do not divide in culture and are in heterogeneous states of neurodifferentiation, whereas PC12 cells undergo uniform differentiation triggered by the addition of nerve growth factor. Thus, primary neurons are problematic for these assessments, but the PC12 line is especially useful (Coecke et al. 2007; Radio et al. 2008). Similarly, because the PC12 line has two defined differentiation end points (acetylcholine and dopamine), it can be readily used to evaluate the potential of test agents to interfere with the appearance of neurotransmitter phenotypes, providing a proof of principle for neurotransmitter switching; this leads the way to examining a wider range of phenotypes *in vivo*.

## Conclusions

Although PAHs can act directly as developmental neurotoxicants, exclusive of endocrine or secondary systemic effects, their impact is greatly modified by coexposure to other neurotoxicants. Accordingly, the effects attributable to PAHs may be quite different depending on which other agents are present and on their concentrations relative to each other. Studies of human populations may thus show different outcomes for PAH effects depending on these other contributors. Here, we selected specific contaminants (dexamethasone, chlorpyrifos, and nicotine) that can be tracked from medical histories or from environmental exposure assessments, an approach that points the way to being able to study these interactions in human populations using existing databases. Indeed, our results reinforce the value of *in vitro* models in evaluating the complex effects of multiple toxicant exposures and in producing testable hypotheses for clinical and epidemiologic studies of human populations.
